# Bayesian Estimation of the Timing and Severity of a Population Bottleneck from Ancient DNA

**DOI:** 10.1371/journal.pgen.0020059

**Published:** 2006-04-21

**Authors:** Yvonne L Chan, Christian N. K Anderson, Elizabeth A Hadly

**Affiliations:** Department of Biological Sciences, Stanford University, Stanford, California, United States of America; University of Chicago, United States of America

## Abstract

In this first application of the approximate Bayesian computation approach using the serial coalescent, we demonstrated the estimation of historical demographic parameters from ancient DNA. We estimated the timing and severity of a population bottleneck in an endemic subterranean rodent, *Ctenomys sociabilis,* over the last 10,000 y from two cave sites in northern Patagonia, Argentina. Understanding population bottlenecks is important in both conservation and evolutionary biology. Conservation implications include the maintenance of genetic variation, inbreeding, fixation of mildly deleterious alleles, and loss of adaptive potential. Evolutionary processes are impacted because of the influence of small populations in founder effects and speciation. We found a decrease from a female effective population size of 95,231 to less than 300 females at 2,890 y before present: a 99.7% decline. Our study demonstrates the persistence of a species depauperate in genetic diversity for at least 2,000 y and has implications for modes of speciation in the incredibly diverse rodent genus *Ctenomys.* Our approach shows promise for determining demographic parameters for other species with ancient and historic samples and demonstrates the power of such an approach using ancient DNA.

## Synopsis

Modern genetic variation can be used to reconstruct past events in a population's history, such as severe population declines (population bottlenecks). However, ancient DNA has the potential to improve our ability to estimate the timing and severity of such events, increasing our understanding of their causes and consequences. The authors apply a method for estimating historical demography, approximate Bayesian computation, to modern and ancient genetic variation sampled over the last 10,000 y, in order to estimate the timing and severity of a population bottleneck in an endemic Patagonian rodent. Their method shows promise for determining demographic parameters for other species with ancient and historic samples and demonstrates the power of such an approach using ancient DNA.

## Introduction

Genealogy-based population genetics (coalescent theory) provides a mathematical framework for modeling underlying processes such as mutation, demography, and genealogical structure [1], making it convenient and powerful for exploring evolutionary processes and demographic events, as well as for providing a statistical framework for data analysis (see review in [2]). For application to ancient DNA, the serial coalescent framework [3] enables multiple temporally spaced sampling points. Furthermore, new computer packages [4,5] enable biologists to simulate genetic data under complex demographic scenarios facilitating parameter estimation.

In this first application of the approximate Bayesian computation (ABC) framework [6] with the serial coalescent [3], we infer the parameters of a plausible demographic history of *Ctenomys sociabilis,* an endemic subterranean rodent, including bottleneck dynamics, from modern and ancient DNA sequence data. The ABC method compares summary statistics computed on the observed data with those computed on simulated datasets derived from models using known parameters of interest. This approach allows estimation of the posterior probability distributions of the parameter, given the observed data, thereby providing a more detailed understanding of the historical demography of this genetically depauperate species.

The colonial tuco-tuco *(C. sociabilis)* is currently listed as threatened by the World Conservation Union [7]. Modern genetic studies [8] across the range of C. sociabilis and an ancient DNA study sampling the last 1,000 y [9] found extremely low genetic variation at cytochrome *b.* These findings were in contrast to the high amount of ancient genetic variation found from 10,000 to 3,000 y before present (ybp) at a second fossil site [8]. In addition to the low amount of variation found at 15 autosomal microsatellite loci [10], these results suggest the possibility of a severe population decline, or population bottleneck, rather than a selective sweep [8]. A quantitative analysis of the timing and severity of the presumed population decline when combined with environmental factors will help clarify the causes of the bottleneck and help understanding of the nonanthropogenic causes of extinction in mammals.

The amount of neutral genetic variation in a species is due to two primary factors: drift, which decreases variability, and mutation, which increases it [11]. A species that experiences a dramatic reduction in size will lose genetic variation as a function of its population size, growth rate, and the duration of the population contraction [12,13]. Traditional methods of detecting bottlenecks [13–16], however, often require polymorphism (but see [17]), which can be a challenge for species such as C. sociabilis that have negligible modern genetic variation [8,10]. As an alternative, combining information regarding modern and ancient levels of variability can yield significant insights into the population dynamics associated with historical changes in genetic variation [18].

We used the serial coalescent to model the demographic history of C. sociabilis over the last 10,000 y and to estimate the timing and severity of the presumed population bottleneck. Most population genetics methods based on coalescent theory have been developed to analyze sequences from a single time point. However, the use of the serial coalescent may prevent some of the unpredictable biases associated with statistical inference and hypothesis-testing resulting from analyzing ancient DNA as a single time point and, furthermore, may increase the statistical power of the methods used [18,19].

First, we began our analysis by testing the null hypothesis of a constant population size for C. sociabilis over the last 10,000 y given our observed recent and ancient variation. We used Serial Simcoal [4] to simulate population genetic data from multiple time points within a serial coalescent framework [3].

Second, we used the serial coalescent with the ABC method in order to estimate the following demographic and marker parameters: the ancient effective population size *(Ne*_*f*__a_), the size of the population following population reduction or bottleneck size *(Ne*_*f*bot_), the time of the population reduction that may have resulted in the loss of genetic variation in the modern populations (t_bot_), the modern effective population size *(Ne*_*f*m_), and the mutation rate (μ). Our demographic model consisted of a constant ancient *Ne_f_*_a_ until the bottleneck time, with an immediate drop in population size to a bottleneck *Ne_f_*_bot_ followed by exponential growth until a modern *Ne_f_*_m_ was reached (Figure 1).

**Figure 1:**
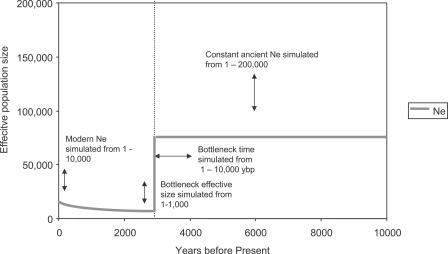
Model of the Population Bottleneck of C. sociabilis Showing a Constant Ancient Female Effective Population Size and Exponential Growth following a Decline in Population Size

Finally, we explored the sensitivity of our results to different types of available data. In order to understand how much our results depended on reasonable prior estimates, we examined different ranges of priors. Furthermore, we investigated alternate modeling scenarios where we estimated the bottleneck parameters with only the modern data or two time points, thereby gaining an understanding of the advantage and utility of ancient DNA for model-based parameter estimation.

## Results

In order to examine the null model of a constant female effective population size for the recent and ancient variation, 1,000 simulations were performed for a range of female effective population sizes (*Ne*_f_) from 100 to 200,000. The average nucleotide diversity was calculated for each simulation, and the 95% confidence hull at each effective population size was plotted against the empirical data to reject the null hypothesis at *p* < 0.05 (Figure 2).

**Figure 2:**
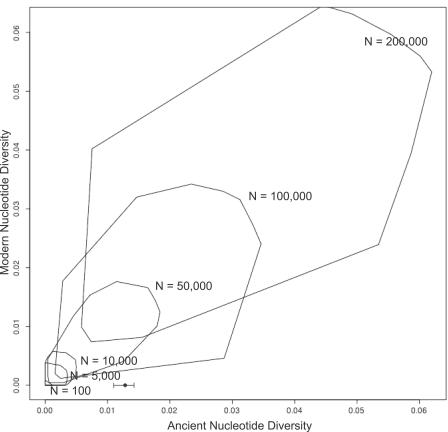
The Observed Ancient and Modern Diversity Plotted below (•) with Standard Error Bars Shows the Rejection of the Null Model of a Constant Population with Sizes Ranging from 100 to 200,000 for the Modern and Ancient Nucleotide Diversity in C. sociabilis Found in Cueva Traful The 95% confidence hulls at each population size assume a single panmictic population based on 1,000 iterations of Serial Simcoal [4] for modern and ancient nucleotide diversity.

The posterior density curves based on 1,000 acceptances out of 1 million iterations (δ = 0.336) for bottleneck time and effective population size, as well as modern and ancient effective population size and mutation rate, are shown in Figure 3. Mean, mode, and quantiles of parameter estimates are given in Table 1. Also shown in Table 1 are the bias, root-mean-square error, and standard error of 1,000 simulated datasets based on the mean estimated parameter values. The posterior density curves for all five parameters contain peaks, indicating that the posterior density curves differ markedly from the uniform priors (Figure 3A–3E). This suggests that the modern and ancient genetic data contained enough information to estimate the demographic and marker parameters.

**Figure 3:**
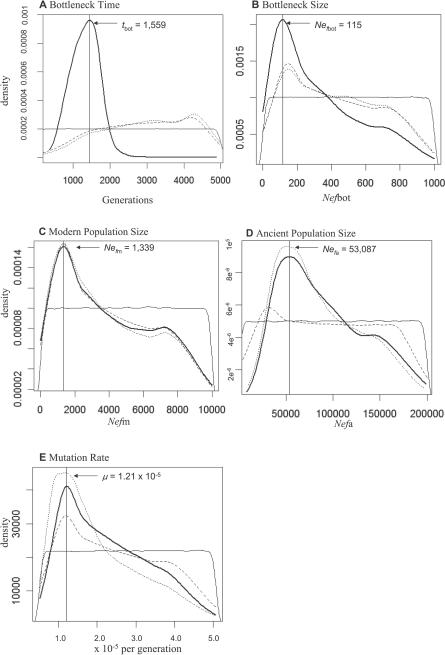
Posterior Density Curves of Model Parameters Based on 1,000 Accepted Values from 1,000,000 Iterations of the Bottleneck Model Thick black line is the posterior density curve of the bottleneck model which includes multiple time points, dashed line is the posterior based on modern data only, and dotted line is the posterior based on two time points, modern and ancient. The mode is indicated with a vertical line and labeled. Thin line is the prior density curve based on 1 million simulated values. (A) Bottleneck time in generations. Assuming a 2-y generation time the peak of the posterior density curve is at 3,118 ybp. Only multiple time points allow reasonable estimation of the bottleneck time. (B) Bottleneck size. (C) Modern population size. (D) Ancient population size. The modern data alone do not provide an estimate the ancient population size; however, with two time points, an estimate of the magnitude of the decline from the ancient population size is possible. (E) Mutation rate expressed as mutations per 253-bp region per generation.

**Table 1:**
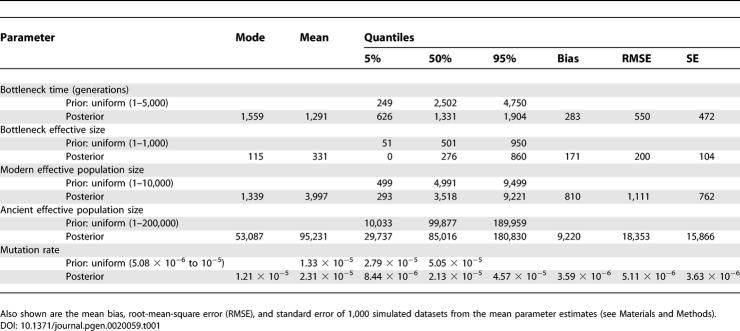
Mode, Mean, and Quantile Values of the Prior and Posteriors for the Demographic and Marker Parameters of C. sociabilis Used in This Study

The highest support was for a mean bottleneck time of 1,291 generations. The posterior density curve for the bottleneck time was particularly sharp (Figure 3A), and the relatively small 95% confidence intervals from 626 to 1,904 generations indicate the highest probability of the bottleneck occurring approximately 2,600 ybp, and probably did not occur at a time less than 1,200 ybp or greater than 4,000 ybp given the genetic data (Table 1).

The estimated bottleneck effective size based on the mean of 331 females was extremely small. Furthermore, the peak of the curve indicates a higher probability of a bottleneck effective size of less than 300 (Figure 3B). Given the estimated ancient effective size of 95,231, this constitutes a dramatic population decline of 99.7%.

The posterior density curves for the modern and ancient effective population size, the mutation rate, and, to a lesser degree, bottleneck size, show relatively high “trailing” tails to the right of the distributions that skew the mean values for each of these parameters (Figure 3B–3E). Although some of the tails may be due to evolutionary variance, the interdependence of each of the parameters on one another probably has a large influence. The posterior density curves represent not only the parameter of interest but also the interaction of all parameters simultaneously. Effective population size strongly depends on mutation rate. Furthermore, when either bottleneck size, modern effective population size, or ancient effective population size is close to zero, the other population size parameters vary more. We were most interested in the time and severity of the bottleneck, and thus the mutation rate and modern effective population size were less critical parameters we incorporated because we lacked sufficient information to set their values in the model.

### Sensitivity of Demographic and Marker Parameters to Different Models and Prior Distributions

In Bayesian analysis, the choice of the prior distribution can have a large impact on the posterior distributions; therefore, we examined the behavior of our parameter estimates to different priors (summarized in Table 2, see Materials and Methods for the choice of prior distributions). The bottleneck effective size and timing of the bottleneck (once the generation time was converted to ybp) were not sensitive to a 2-y versus a 1-y generation time (*Ne_f_*_bot_ = 331 versus 384, and *t*_bot_ = 2,582 versus 2,483 ybp), although the ancient effective population size was 33% larger in the 1-y scenario. None of the parameters changed appreciably when we ran the same demographic model with a fixed mutation rate equivalent to 5% per million years (253-bp region rate per generation = 2.03 × 10^−5^) instead of a variable mutation rate (*Ne_f_*_bot_ = 342 versus 331, *t*_bot_ = 1,350 versus 1,291 generations, *Ne_f_*_a_ = 97,824 versus 95,231, *Ne_f_*_m_ = 3,635 versus 3,997). Increasing the prior for the modern effective population size from 1 to 10,000 to 1 to 100,000 demonstrated the insensitivity of the parameter estimates to the modern effective population size. Although the bottleneck size decreased slightly (*Ne_f_*_bot_ = 243 versus 331), the ancient size and bottleneck time changed little (*t*_bot_ = 1,299 versus 1,291 generations, *Ne_f_*_a_ = 98,997 versus 95,231), even though the model estimated an unrealistically high modern effective population size (*Ne_f_*_m_ = 41,162) given what we know about the census size of the modern species. Finally, changing the prior for the bottleneck effective size had little impact on the bottleneck time, ancient effective population size, or the mutation rate (*t*_bot_ = 1,906 versus 1,291, *Ne_f_*_a_ = 94,639 versus 95,231, μ = 2.26 × 10^−5^ versus 2.31 × 10^−5^). However, the bottleneck effective size and the modern effective size were higher and lower, respectively (*Ne_f_*_bot_ = 3,487 versus 331, *Ne_f_*_m_ = 2,464 versus 3,997), indicating that the bottleneck effective size is not as robust an estimate as the bottleneck time.

**Table 2:**
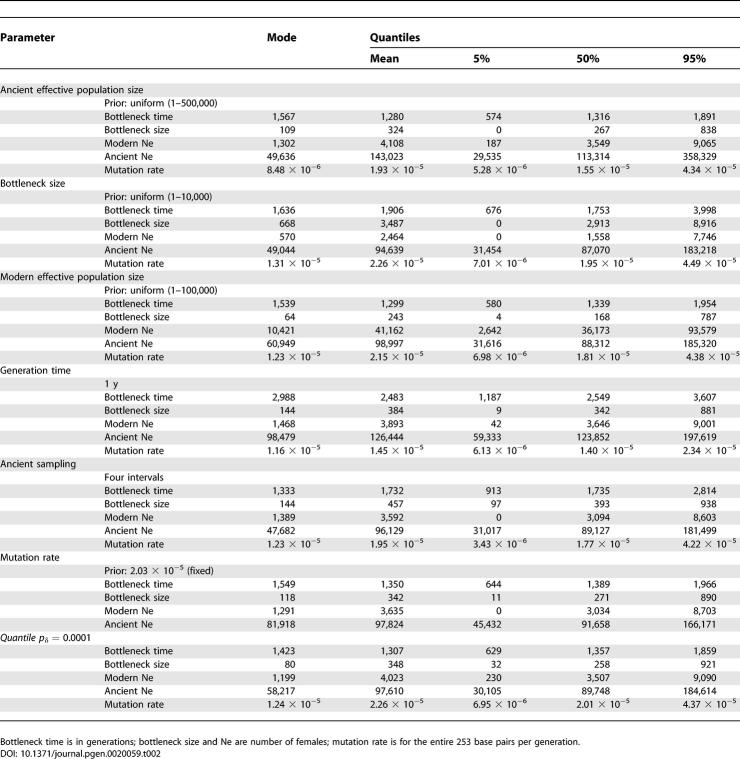
Simulation Results Testing the Sensitivity of Demographic Inferences to Changes in Marker and Demographic Priors for C. sociabilis over 10,000 y

We also examined the sensitivity of the parameter estimates to two and four sampling intervals, instead of three. We found that with two sampling intervals, our method was less able to resolve the mutation rate and ancient effective population size. This approach yielded broad posterior distributions, without well-defined peaks. With four sampling intervals, we found that the parameter estimates did not really change (*Ne_f_*_bot_ = 457 versus 331, *t*_bot_ = 1,732 versus 1,291 generations, *Ne_f_*_a_ = 96,129 versus 95,231, *Ne_f_*_m_ = 3,592 versus 3,997, *μ* = 1.95 × 10^−5^ versus 2.31 × 10^−5^).

Finally, an examination of the sensitivity of our results to the choice of delta showed little change among the estimates (*Ne_f_*_bot_ = 348 versus 331, *t*_bot_ = 1,307 versus 1,291 generations, *Ne_f_*_a_ = 97,610 versus 95,231, *Ne_f_*_m_ = 4,023 versus 3,997, *μ* = 2.26 × 10^−5^ versus 2.31 × 10^−5^).

### Alternate Modeling Scenarios

To examine the importance of utilizing ancient DNA data from multiple time points, we performed the same analysis with just the modern data and with only two time points (a modern sample and an ancient sample 10,000 ybp; results are summarized in Table 3). The posterior density curves are plotted in Figure 3 with the curves from the complete dataset. The peaks of the posterior density curves indicate that the modern data and two–time point data are sufficient to provide reasonable estimates of the bottleneck size and modern population size. Furthermore, two time points enabled estimation of the ancient population size and mutation rate. However, the broad distributions for the bottleneck time indicate that ancient DNA from multiple time points is necessary to estimate the timing of the bottleneck.

**Table 3:**
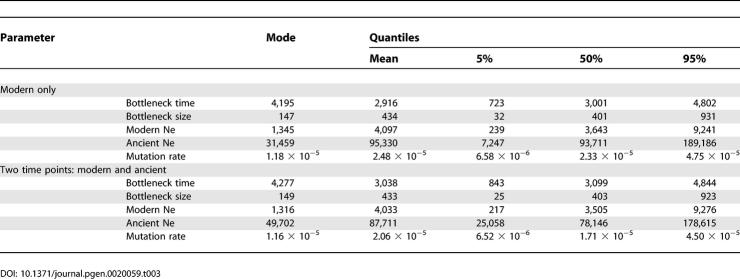
Mode, Mean, and Quantile Values of the Prior and Posteriors for the Demographic and Marker Parameters of the Modern Only and Two–Time Points Analysis

## Discussion

C. sociabilis has undergone a significant change in genetic diversity sometime during the last several thousand years. We are able to reject the null hypothesis that the change in genetic diversity occurred under a constant population size during this time. Instead, we attribute this change to a severe reduction in population size. In this application of the ABC method with the serial coalescent we found there is enough information in ancient DNA from multiple time points to estimate the timing and severity of the population bottleneck. Although the bottleneck time was particularly well estimated with our ancient data, the remaining parameters, bottleneck size, modern effective population size, ancient effective population size, and mutation rate were less so. This study is based on and restricted to mitochondrial DNA, effectively a single linked locus. Because of its presence in high copy number, mitochondrial DNA has been heavily relied upon for ancient DNA studies; however, multiple unlinked loci would improve this estimation procedure. Despite this limitation and although we have no ancient DNA samples from 850 ybp to 3,292 ybp, this analysis indicates that the bottleneck was likely prior to 850 ybp. Our results demonstrate that C. sociabilis has likely persisted with low genetic variation for over 2,000 y.

Estimates of a large ancient effective population size prior to the bottleneck event followed by a decline of almost 100% in effective population size shows that even rodent species can suffer large nonanthropogenic declines. Although we do not know the cause of the population decline and the associated loss of genetic diversity, a few possibilities have emerged. These include a volcanic eruption during a period of environmental change and competition from other ctenomyid species [8]. A similar decline in population size resulting from a 1988 volcanic eruption was observed in another ctenomyid species, *C. maulinus brunneus* where population censuses over several years documented a 91.3% decline [20]. However, C. sociabilis is unique because very little modern genetic variation has been detected despite sampling populations across the entire range of the species [8]. Therefore, the factors that were responsible for the near extinction of this species left it with little genetic variation.

Evidence of a severe population decline in C. sociabilis and persistence with low genetic variation has evolutionary implications for the mode of speciation in this genus*.* The influence of population bottlenecks on genetic variability has important evolutionary implications because of the involvement of sudden shifts in genetic variation on founder effects and speciation [21]. *Ctenomys* is extremely speciose (>56 species) and differentiated in the Plio-Pleistocene. It is therefore one of the most rapidly diversifying groups of mammals [22,23]. Due to the large amount of karyotypic variation (2n = 10 to 70 [22]), it has been proposed that karyotypic evolution is a driver of speciation in this group. An interesting consequence to this mode of speciation is that it implies a founder event and severe population size reduction coincident with cladogenesis, with reduced hybrid fitness while the species is recovering. Our study demonstrates a ctenomyid species can persist through near extinction with low genetic diversity over millennia.

Factors that may reduce the rate of recovery of variation in either a nearly eradicated population or a newly established founding population of small size are the same and include breeding structure and low dispersal in fragmented habitats. Low variability resulting from a bottleneck is predicted to increase the detrimental effects of inbreeding, fixation of mildly deleterious alleles, and loss of adaptive potential [24]. Although other species, such as the Golden Monkey, have persisted with low genetic variation following a nonanthropogenic decline [17], given the severity of the bottleneck in *C. sociabilis,* how has it persisted and recovered over the last 1,000 y?

C. sociabilis currently possesses an unusual social system for the genus *Ctenomys* in that it is the only species that is wholly social [25]. Following a bottleneck the population experiences a decrease in genetic diversity which may induce new selective trends by changing the internal genetic environment [20]. One hypothesis is that sociality may have evolved as a result of this bottleneck, as has been suggested in other species such as the Argentine ant [26]. This hypothesis postulates that the bottleneck results in increased relatedness among individuals, increasing the benefits of philopatry, while the costs of dispersal remain high. Once the barriers to territoriality have broken down, this social system spreads throughout the species. The influence of this social system, either as a cause or consequence of the bottleneck, will be interesting to explore with future studies of this species.

Although we modeled this species as a single panmictic population, given the typical biology of subterranean rodents [27] it is more likely that this species is structured both geographically and demographically. If the bottleneck was the result of a range contraction and the persistence of a few peripheral populations, and if the species was not already social, sociality may have been associated with the peripheral populations before the species contraction. For example, C. rionegrensis and C. dorbignyi populations are mostly solitary; however, one isolated population of each species exhibits a colonial life with collective burrows [22]. In *C. sociabilis,* the southern part of the range could have contained a peripheral population (or populations) existing with limited genetic diversity and differentiating independently from the genetically diverse populations in the northern part of its range. Further modeling of structured populations should provide a more detailed understanding of the historical demography of C. sociabilis.

One caveat is that we use serial coalescent modeling to estimate the female effective population size of C. sociabilis over the last 10,000 y. Since this study is based on mtDNA, our results reflect only the maternal dynamics of this species. Additionally, mtDNA is a single nonrecombining locus and therefore subject to stochastic variation and to sampling variance. It is possible that the lack of variation observed in cytochrome *b* is a result of a selective sweep either at cytochrome *b* or somewhere else in the genome. However, we think this is unlikely due to the limited amount of variation at autosomal microsatellites in modern populations as well [10]. Use of ancient DNA may overestimate diversity due to damage to the DNA template. Precautions have been taken to avoid considering DNA damage as true variation; including separate reamplification of 66% of the samples to detect cytosine deamination [28], overlapping amplified fragments, and cloning [8].

In conclusion, we have successfully applied Bayesian techniques to ancient DNA to demonstrate the persistence of an endemic species for 2,000 y, despite its near extinction. In addition to our application of a serial coalescent model, the novelty of our approach is that we use multiple time points to bracket a bottleneck with the goal of determining multiple demographic parameters. Our approach is particularly useful when modern genetic variation is insufficient to reconstruct historical demography of a species.

Our method can be applied to temporal data from ancient DNA and museum skin studies and will be more powerful when multiple time points are sampled. We demonstrate the robustness of our results to different ranges of priors indicating that reasonable estimates may be obtained even without a large amount of prior knowledge. Additionally, our alternate modeling scenarios show that with only the modern data, estimates of the modern population size and bottleneck size are possible. However, a single ancient sample allows estimation of the ancient population size providing information on the severity of the population decline. But having ancient DNA from multiple time points is necessary to provide estimates of the timing of the bottleneck.

An important consideration for conservation of endangered species is whether low variation is a cause of rarity, or whether rarity is the cause of low variation. Proper design of conservation and management programs for endangered species hinges on an understanding of the species' evolutionary history and genetic status. This information is necessary for designation of management units as well as for the development of strategies to prevent loss of variation due to drift [29]. Our results reveal how ancient DNA can play a critical role by providing knowledge of historical demography and diversity of species at risk.

## Materials and Methods

### Study sites and sampling.

Genetic sequences used in this analysis were obtained from two excavation sites: Estancia Nahuel Huapi locality 1 [9] (ENH; *n* = 14 genetic samples), and Cueva Traful (CT) [30] (*n* = 33 genetic samples) (Figure 4). ENH is located 20 km northeast of San Carlos de Bariloche, Neuquén Province, Argentina, near the center of the modern geographic range of *C. sociabilis*. The excavation site is a 1 m × 1 m pit beneath an overhang of rhyolitic volcanic bedrock that served as an owl roost. It contained nine naturally stratified units to a maximum depth of 81 cm below datum. Radiocarbon age calibration of the deposits indicated a maximum age of 1,000 y [9]. DNA was extracted from dental material and 253 bp of mitochondrial cytochrome *b* amplified as described in Hadly et al. [9]. Fourteen of the ENH sequences spanning 1,000 ybp to the present [9] were included in this analysis (see Figure 4 for sample sizes at each unit). Only one haplotype was identified (segregating sites = 0, nucleotide diversity = 0.0000); this haplotype also matched 53 modern C. sociabilis sampled from six populations across the current range previously published in Chan et al. [8].

**Figure 4:**
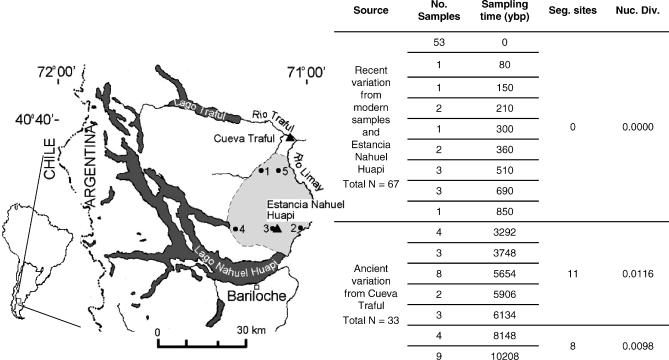
Map of Sampling Sites and Modern Range of C. sociabilis with Table of Temporal Sampling Listed are number of samples per sampling time and observed values for segregating sites and nucleotide diversity used in the bottleneck model. Numbers on map indicate locations of modern populations [8].

CT is an archeological site and owl roost located 30 km north of ENH near the confluence of the Traful and Limay rivers (Figure 4). Due to the deposition of owl pellets through time, the cave provides a temporal sequence of small mammal skeletal material radiocarbon dated to a maximum age of 10,209 ± 96 ybp [8]. CT is not within the northern limit of the current C. sociabilis range (Figure 4); however, morphological evidence suggested that C. sociabilis was present more than 3,000 ybp near CT [31]. DNA was extracted from teeth following the protocol of Hadly et al. [9]. Thirty-three sequences phylogenetically identified as C. sociabilis from the same 253 bp of cytochrome *b* amplified from the ENH samples were included in this analysis from eight stratigraphic units ranging from 10,209 ± 96 to 3,293 ± 49 ybp (Figure 4). In contrast to the negligible recent genetic variation, eight haplotypes were found in CT. Those haplotypes fell into two clades that overlap temporally in CT: a modern clade consisting of two haplotypes representing nine individuals, one of which exactly matched the recent haplotype, and six haplotypes representing 24 individuals that belonged to a divergent ancient clade (total uncorrected sequence divergence = 4.3%). Sequences can be found under GenBank accession numbers DQ402060 to DQ402066. Of 11 polymorphic sites, eight changes were synonymous and three were nonsynonymous (nucleotide diversity = 0.01283 ± 0.00167, haplotypic diversity = 0.71 ± 0.06, *n* = 33) [8].

The possibility of the ancient clade belonging to an extinct sibling species cannot be excluded. Although we think this is unlikely for the reasons outlined in Chan et al. [8] (supplemental online material) and for the following reasons: (1) there are no morphological differences among the teeth belonging to the two clades; (2) the amount of polymorphism within C. sociabilis is comparable to other ctenomyid species; (3) the level of divergence is at the low end for sister taxa in *Ctenomys;* (4) while the average interclade sequence divergence (3.52%) is not trivial, it is not unusual neither within mammals, nor subterranean rodents, nor within *Ctenomys* in particular; and (5) it would be highly unusual for such closely related sibling species to co-occur without interbreeding.

### Estimation procedure for demographic parameters.

The lack of recent genetic variation from approximately 1,000 ybp to present at cytochrome *b* and the low genetic diversity in a modern population at 15 microsatellite loci [10] in combination with the large amount of ancient variation found from approximately 3,000 to 10,000 ybp suggests the possibility of a large decrease in population size (i.e., population bottleneck) at some point in the history of C. sociabilis. Therefore, we used the amount of genetic variability at the mitochondrial locus cytochrome *b* in C. sociabilis to estimate the demographic history of the female effective population size over time.

### Prior distributions of demographic and marker parameters.

In the Bayesian analysis, the “prior” is based on previous knowledge of the biotic system. Information from the literature was used to derive the values for demographic parameters as well as the prior distributions of demographic and marker parameters used in the models as follows. Generation time was set at 2 y. Individuals of C. sociabilis reach sexual maturity at about 9 mo, produce one litter a year, and can live up to 4 y. The observed mean number of female generations in one study population was 2.0 ± 1.0 (range 1–4; *n* = 19 social units) [32,33]. The effect of assuming a generation time of 1 versus 2 y was investigated in a sensitivity analysis.

We used a finite-sites mutation model based on results derived from Modeltest v. 3.7 [34] from an analysis that included the data used in this study and all available cytochrome *b* sequences from GenBank. Using Akaike information criterion, an HKY + I + G model [35] was selected with a transition/transversion ratio = 6.62, proportion of invariable sites = 0.4138, and gamma distribution shape parameter = 0.8423. A uniform prior distribution for the mutation rate, μ from 1% to 10% of the region per Myr (5.08 × 10^−6^ to 5.08 × 10^−5^ for the 253-bp region per generation) was based on conservatively low and high estimates for the mutation rate at cytochrome *b* in mammals [36,37]. We investigated the sensitivity of our mutation rate to a variable rate versus a set mutation rate.

The prior for the modern effective population size was chosen according to an estimated current census population size of less than 10,000 adults (E. A. Lacey and J. R. Wiezcorek, personal communication, December 2004). To be conservative, since the model is based on the female effective population size, the prior for the modern effective population size was a uniform distribution from 1 to 10,000 haploid individuals. Sensitivity of our results to larger *Ne_f_*_m_ was also examined.

The binning method for samples that is used to calculate summary statistics may also have an influence on the posterior distributions. We chose three time intervals—one representing the recent variation and two representing the ancient variation. A small number of bins increases the sample size for each summary statistic; however, information is lost as samples from multiple time periods are grouped together. As the number of summary statistics calculated increases, errors are increased and more iterations are needed. We investigated the sensitivity of our results to the number of time intervals by examining the results with two and four time intervals as well.

Finally, since we are examining the demographic history over the last 10,000 y we chose a uniform prior distribution for the bottleneck time from 1 to 10,000 ybp. The demographic history of C. sociabilis prior to 10,000 ybp is beyond the scope of this study, and likely encompasses very large environmental perturbations as it spans the glacial-interglacial transition at the terminal Pleistocene.

### Alternate modeling scenarios.

In order to understand the importance of sampling ancient DNA from multiple time points, we investigated two alternate modeling scenarios. The first one represented a single time point consisting of the 53 modern monomorphic samples from across the species range, similar to a study that does not incorporate ancient DNA. The second was meant to represent a study that includes ancient DNA, but does not have adequate dating. This modeling scenario consisted of two time points, the recent variation (53 modern samples) and ancient (which contained 47 samples from ENH and CT all dated at 10,208 ybp).

### Procedure for parameter estimation.

A full description of the ABC method is given in [6]. Bayes' theorem can be approached from a sampling-resampling perspective [38] where the empirical data are used to approximate the distribution of the parameter of interest. Previous studies have utilized this technique for estimating complex demographic histories from modern DNA [6,39,40] but we applied this approach to the estimation of demographic history from ancient DNA using the serial coalescent. Within the Bayesian framework, the demographic model specifies the prior distribution of the genealogical tree and parameter estimates are inferred from the posterior distribution of the genealogical tree given the observed data, the coalescent prior for the genealogy, and the priors for the demographic parameters. With this method, parameter estimation is achieved by comparing a summary statistic calculated from the empirical data with the distribution generated by Monte Carlo simulations of the coalescent process. In particular, we employed the ABC approach specified by Beaumont et al. [6] which uses a rejection-sampling method for simulating an approximate posterior distribution and also employs smooth weighting and linear adjustment [6]. One advantage of the ABC method is its insensitivity to the choice of δ. However, we examined the influence of δ on our analysis by performing 10 million iterations and accepting the quantile *p*_δ_ = 0.0001.

In this study, rejection-based approximate Bayesian inference methods were implemented using Serial Simcoal [4] and the statistical package, R version 2.0.1. The use of Serial Simcoal in this paper differs from previous applications of the serial coalescent to ancient data [17] in that multiple time points are incorporated into a single tree (instead of being limited to two time points). Summary statistics for the observed data, nucleotide diversity and segregating sites, were calculated using DnaSP [41]. Statistics for the modern samples were taken from Chan et al. [6].

Genetic variation in the recent and ancient samples was summarized by six statistics, calculated as the number of segregating sites [42] and the average nucleotide diversity per site [43] for each of three time periods: (1) modern and recent nucleotide diversity and segregating sites (*S*_m_, π_m_); (2) ancient nucleotide diversity and segregating sites from approximately 5,000 to 3,000 ybp (*S*_a1_, π_a1_); and (3) ancient nucleotide diversity and segregating sites from approximately 10,000 to 5,000 ybp (*S*_a2_, π_a2_). We chose the number of segregating sites and nucleotide diversity because segregating sites reflects the amount of variation in a sequence but is not influenced by nucleotide frequencies, and nucleotide diversity is affected by nucleotide frequencies. Under a neutral model of evolution with constant population size these two statistics should be the same. The difference between these statistics is Tajima's D [44] which is a commonly used to test deviations from the neutral model such as selection, population structure, or changes in population size.

Posterior distributions for the estimated parameters were estimated using the following algorithm:

Simulate a dataset using the serial coalescent process and demographic and marker parameters (*Ne_f_*_bot_, *t*_bot_, *Ne_f_*_a_, *Ne_f_*_m_, μ) drawn from uniform prior distributions.

Compute summary statistics for the simulated dataset (*S*_m_*, π_m_*, *S*_a1_*, π_a1_*, *S*_a2_*, π_a2_*) and record summary statistics and parameter values.

Repeat 1 and 2 until 1 million simulations are performed.

Compute summary statistics (*S*_m_, π_m_, *S*_a1_, π_a1_, *S*_a2_, π_a2_) from the observed dataset.

Calculate the normalized Euclidean error using the following formula:


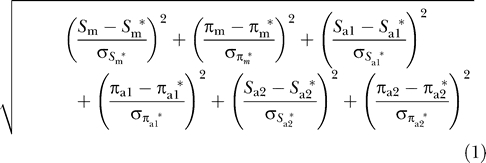


The values for *Ne_f_*_bot_, *t*_bot_, *Ne_f_*_a_, *Ne_f_*_m_, and μ for the 1,000 (quantile *p*_δ_ = 0.001) datasets with the smallest Euclidean distances were recorded for the posterior distributions. The remaining datasets were rejected [39].

Local linear regression adjustment and smooth weighting of 1,000 acceptances was implemented as described in [6] using the functions lm() and Locfit in R version 2.0.1 to estimate the posterior density function. Summary statistics were calculated using the summary and quantile functions, and the location of the mode was estimated from a kernel density estimate using the Locfit package. Bias and root-mean-square error were calculated from the mean values estimated from 1,000 datasets that were simulated with the mean parameters estimated by the bottleneck model. For computational efficiency, the same 1 million simulations were bootstrap resampled to derive the 1,000 acceptances for each of the 1,000 simulated datasets.

Caution must be applied when interpreting estimates of effective population size based on models with certain assumptions such as a single panmictic population across the species or utilizing a particular demographic model. One problem with the model is that the samples from CT could have consisted of multiple populations. In order to investigate the influence of sampling over structured populations, we ran multiple population simulations and binned all the samples to see what effect calculating a single summary statistic for multiple populations would have on genetic diversity. As expected, we found that population structure increases the expected amount of genetic variation in the overall sample (unpublished data). This would result in an overestimation of the ancient effective population size. However, even with very low migration rates among several populations, a large decline in population size is necessary to lose all genetic variation in the species.

## Supporting Information

### Accession Numbers

Sequences used in the study can be found under GenBank (http://www.ncbi.nlm.nih.gov/Genbank) accession numbers DQ402060–DQ402066.

## Abbreviation

ABC: approximate Bayesian computation
ybp: years before present
